# Fecal incontinence and rectal prolapse

**DOI:** 10.1007/s12664-020-01014-1

**Published:** 2020-01-30

**Authors:** Naveen Kumar, Devinder Kumar

**Affiliations:** 1 Anatomy Fellow, Queen Mary University of London, London, UK; 2 Professor of Gastrointestinal Surgery, St George’s University of London, London, UK

Fecal incontinence is the involuntary loss of flatus, liquid, or solid stool from the anus. It is more common in women than men. The prevalence of fecal incontinence increases with advancing age. It is either idiopathic or due to injury to the sphincter complex. In women, obstetric trauma is the commonest cause of anal sphincter injury leading to incontinence. Other causes include iatrogenic sphincter injury during hemorrhoidectomy and fistula surgery and acute trauma to the perineum. Overt sphincter damage due to third- or fourth-degree vaginal tears during childbirth has been reported in up to 0.7% of all vaginal deliveries resulting in symptomatic fecal incontinence [1, 2]. The pudendal nerve can also be damaged during childbirth trauma resulting in late development of fecal incontinence [3]. Some patients sustain both mechanical and neurological trauma to the sphincter complex [4]. Risk factors associated with sphincter injury include forceps delivery, primiparous mother, birth weight of the baby > 4 kg, and occipito-posterior presentation. A posterolateral episiotomy does not protect against a sphincter tear [5]. Anal endosonography has enabled accurate imaging of the sphincter complex resulting in accurate recognition of occult anal sphincter defects [6–8]. In one study of 62 women with a history of obstetric trauma, endoanal ultrasonography revealed an external anal sphincter defect in 90% and an internal sphincter defect in 65% of patients [9]. In 8 of the 10 women who had a forceps-assisted delivery, there was evidence of sphincter damage.

In another study [10], 127 women who had a vaginal delivery underwent endoanal ultrasonography postpartum. Of the 79 primiparous mothers who had intact sphincters before childbirth, 28 showed damage to one or both sphincter muscles following delivery. The extent of the damage to the sphincter complex was also mapped accurately by endoanal sonography. All the external sphincter defects were located anteriorly.

Conservative management strategies in the form of dietary manipulation, bowel retraining, and lifestyle measures form the mainstay of treatment of idiopathic incontinence. For some patients, posterior tibial nerve stimulation (PTNS) or anal irrigation may be required to treat the symptom of incontinence. When all else fails, in selected patients, a stoma may be required to treat incontinence and improve the quality of life. A comprehensive review of the entire spectrum of fecal incontinence is beyond the scope of this article, so we have decided to focus on only one important factor, namely, obstetric anal sphincter damage, in relation to the management of fecal incontinence. The standard treatment of obstetric anal sphincter injury is a primary repair. Following a primary repair, up to a third of all patients have a poor outcome, and these patients require further assessment and a secondary repair. Long-term functional results of sphincter repair remain suboptimal. In a recent study [11] of 255 eligible patients with nearly two decades of follow up, only a few women were continent. Advanced age at the time of repair, repeated repairs, and menopause duration longer than 5 years were associated with a poor outcome.

## Assessment

**Fig. 1:**
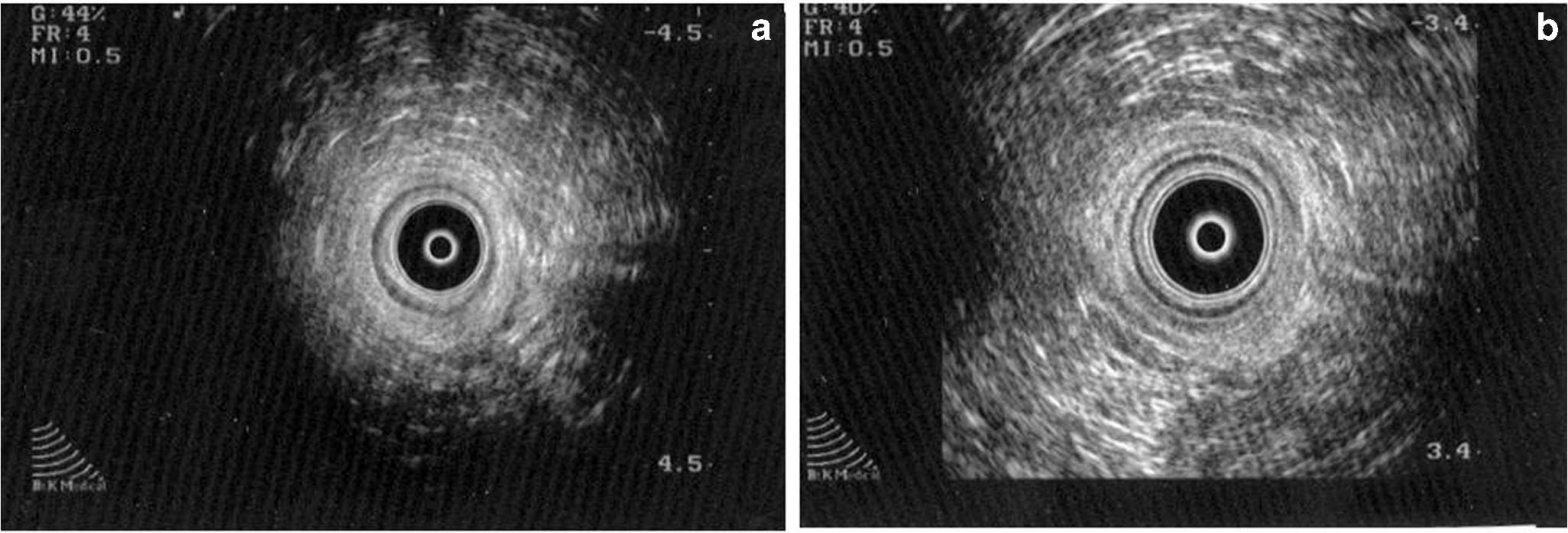
**a** Showing a normal sphincter complex on endoanal ultrasound. **b** Showing an anterior external sphincter defect following childbirth trauma. The internal sphincter is intact

Accurate assessment of the extent and severity of damage after delivery is crucial to proper planning of a future treatment strategy. It consists of assessment of function mainly through symptoms, anatomical structure by using endoanal ultrasonography (Fig. 1a, b), and physiological assessment using anorectal manometry, which measures resting and squeeze pressures generated by the internal and external sphincter muscles, respectively. In addition, volume to first sensation and maximum tolerable volume along with the rectoanal inhibitory reflex are measured. The latter gives information on the integrity of the neurogenic mechanisms.

Some authors have advocated the use of endoanal ultrasound in the labor ward [8]. In a systematic review, Walsh and McGrivell [12] concluded that from the available data, results are consistent with improvement in severe anal incontinence with the use of endoanal ultrasound as an adjunct to clinical examination prior to perineal repair in primiparous women both at ≥ 6 months and > 6 months postpartum. In contrast, endoanal ultrasound was shown to be associated with an increase in women’s perineal pain at the 3-month time point. Since this evidence is based on only one trial, which only followed women for 12 months, more evidence is needed in order to confirm or refute these findings.

It has also been reported that on clinical examination alone, third- and fourth-degree tears are overdiagnosed in the labor ward [13]. In a retrospective review of prospectively collected data over a 10 year period from 1495 patients who had had a primary repair for sphincter injury, Thomas et al. [13] found that endoanal ultrasonography demonstrated residual sphincter defect in 792 (53%) and normal sphincters, with no evidence of repair, in 661 (44%) patients. The majority of injuries involved both the external and internal sphincters (*n* = 501). Significant reductions in resting and squeeze pressures were seen when those with a sphincter defect were compared to those with intact sphincters. However, there was no significant difference in the mean Cleveland Clinic fecal incontinence scores. They concluded that third- and fourth-degree tears appear to be overdiagnosed. Primary repair appears to be unsuccessful in the majority of cases, and there appears to be a poor correlation between objective and subjective assessment of sphincter function. These findings have important implications for the management of such patients. In this series, 44% had no evidence of a sphincter repair or defect, and if these patients on the basis of a clinical diagnosis are subjected to a secondary repair, the result is going to be less than satisfactory. Equally, it points to an additional mechanism other than the sphincter complex for the incontinence.

Chandra et al. [14] have utilized the concept of on-table assessment of the sphincter complex before and after the repair to ensure that the repair was complete. They did not include patients who had had a previous repair. A Doppler study was also included to ascertain the integrity of the neurovascular bundle. Although conceptually their approach seems logical, at 62 months follow up, there was no significant difference in terms of function between the two groups.

## Surgical technique and outcomes

Primary repair in most women who sustain a third-degree tear is inadequate [5]. The majority of these women have residual sphincter defects, which are thought to be mechanical disruption rather than pudendal nerve damage [5]. Repair of the sphincter defects is performed either by using the end-to-end method in which the sphincter ends are dissected and then approximated using absorbable or nonabsorbable sutures or by overlapping the sphincter ends and holding them in place using monofilament sutures. Proponents of the overlap repair claim that it provides superior results. Chandra et al. [14] in this issue of the *journal* have proposed an ultrasound-guided technique of sphincteroplasty, in which not only the integrity of the mechanical component of the repair but also the neurovascular bundle can be checked at the end of the procedure. The argument put forward in its favor is that the deteriorating results over time are due to compromised neurovascular supply or an incomplete repair. The procedure is performed in the lithotomy position under general anesthesia. We recommend that the dissection is started laterally rather than in the midline as it makes it easier to enter the right plane of dissection and also reduces the blood loss. Once the operator is in the right plane of dissection, it is easy to find the sphincter muscle ends. We routinely perform a levatorplasty as it helps to make the overlap repair tension free and also repairs any undetected sphincter damage in the upper part of the sphincter complex. Immediate and long-term results following anal sphincter repair have been a persistent topic of debate. It has been reported that up to 65% of patients maintain good results in 3–10 years following sphincter repair [15–20]. Berg et al. in a study of 94 patients utilized separate suturing of the internal and external sphincter defects and reported that two third of the patients maintained symptomatic improvement at least 3 years after repair. In a systematic review comparing the results of end-to-end sphincteroplasty with overlap repair, it was found that the overlap repair confers a slight benefit in the short-term for fecal urgency, but at 3 years, there was no significant difference in the results of the two techniques [21]. In the first year, the anal incontinence score for the overlap repair also improved, but at 3 years, it did not significantly differ from the end-to-end repair [21].

We conclude that the anal continence mechanism is complex involving the sphincters, rectal function, and stool consistency. Following childbirth, sphincter disruption is common, but a large proportion of these defects are occult and do not cause the symptoms of fecal urgency or incontinence. Endoanal ultrasonography has revolutionized sphincter assessment after childbirth. Sphincter assessment with magnetic resonance imaging (MRI) is feasible but time consuming and expensive and has a long learning curve. Primary repair of sphincter defects results in an unsatisfactory outcome with up to two thirds of patients still showing a persistent defect. Secondary repair of the sphincter muscles is done either through approximation of the disrupted sphincter ends or an overlap repair. In the short-term (1 year), results of the overlap repair are better than those of the end-to-end repair, but at 3 years, there is no significant difference in the outcome between the two types of repair. In this issue of the *journal*, Chandra et al. [14] have utilized the concept of ultrasound-guided sphincteroplasty, but unfortunately, it failed to show any improvement in the results at least in the short-term.

It is intriguing that some patients with sphincter disruption become symptomatic immediately, whereas others with similar injuries may remain symptom-free for decades. We believe that the explanation for this lies in the fact that continence is maintained by a number of factors and sphincter integrity is only one of these. When more than one factor is compromised (i.e. rectal function, stool consistency, and sphincter integrity), it manifests itself as fecal incontinence. Suboptimal functional results following sphincteroplasty may just be a reflection of the fact that we rely on addressing only one of the factors, which is under the control of the operating surgeon, and ignore involving the patient to manage the other factors, which are perhaps just as important.

## Rectal prolapse

**Fig. 2:**
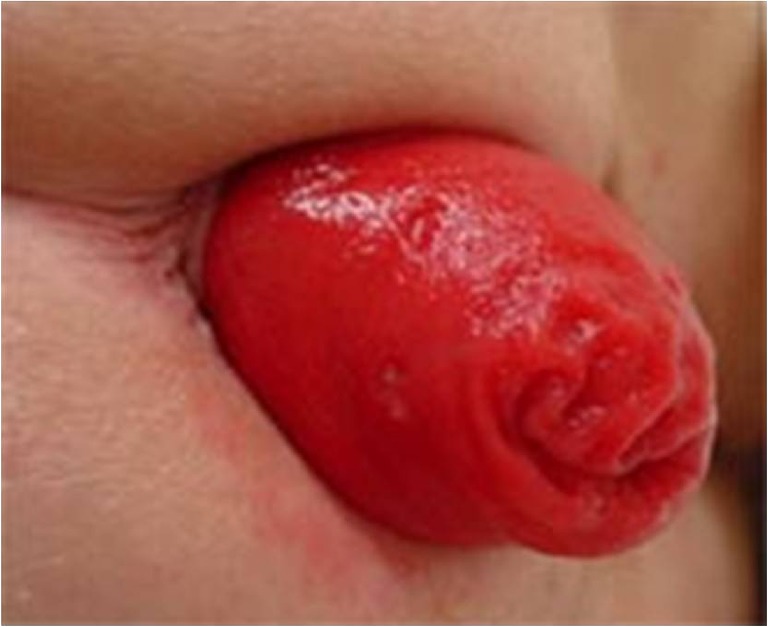
Photograph showing rectal prolapse

Rectal prolapse is the protrusion of full thickness of rectal wall through the anus (Fig. 2). This is called an external rectal prolapse. It affects 2.5 per 100,000 people every year in the UK [22] and is more common in women [23]. Risk factors include traumatic vaginal delivery, multiple vaginal deliveries, straining, and old age [24]. In the internal rectal prolapse, there is intussusception of the rectum above the sphincter complex. Internal prolapse presents as constipation or obstructed defecation in most patients. No specific test is necessary to diagnose or assess the external full thickness prolapse except for patients who have associated fecal incontinence. For internal prolapse, dynamic assessment using barium, isotope, or magnetic resonance (MR) defecography may be helpful.

Surgery is the only definitive treatment of a full thickness prolapse and can provide complete resolution of symptoms. The choice of surgical procedure to a large extent depends on the fitness and age of the patient. In the elderly and frail patient, an anal encirclement procedure may be sufficient. In the fit younger patient, some form of rectal fixation with or without sigmoid resection may be required.

## Surgical options, technique, and outcome

Surgical options include abdominal operations in which the rectum is mobilized down to the pelvic floor with or without dividing the lateral ligaments, and the rectum is then fixed to the sacral promontory with the help of sutures or some form of mesh. The abdominal operations can either be done as open surgery, laparoscopically, or robotically. The other option is perineal surgery, which is traditionally reserved for patients who are deemed unsuitable for abdominal operations, such as the frail and elderly and those who are relatively unfit medically. The procedures normally consist of either a Delorme’s operation or a perineal proctosigmoidectomy. In some patients who are unfit, an anal encircling procedure to stop the rectum from prolapsing out is performed. The third option is to perform excision of the redundant rectum or sigmoid colon with a colorectal anastomosis. This approach is commonly used in patients who have a pre-existing history of constipation. Current evidence shows that there is little difference in outcomes in people undergoing different operations [25].

Much comment has been made on the extent of rectal mobilization during the rectopexy. It is generally accepted that the dissection should continue down to the pelvic floor to minimize the risk of recurrence. Opinions differ regarding the division of the lateral ligaments. Opponents of division of the lateral ligaments believe that it leads to a greater risk of developing constipation postoperatively. Similarly, some authors believe that lateral dissection increases the risk of complications as it has the potential to damage the autonomic nerves running along the lateral aspect of the rectum. Also, lateral dissection increases the risk of developing sigmoidoceles and enteroceles in the future.

We recommend that the lateral peritoneum should be closed after the rectum has been fixed to reduce the risk of such complications. Also, there is a risk of causing rectal kinking if the rectopexy is too tight and pushes the rectum too far back in the sacral hollow. We suggest that the rectum should be carefully checked and if there is an anterior kink then a longitudinal incision anteriorly in the peritoneal covering will release it. In our experience, it helps to alleviate the mechanical reason for postoperative defecatory difficulty.

To overcome the problem of postoperative constipation and slow recovery, the concept of ventral rectopexy has been developed [26]. It has the advantage that it can be performed for both external as well as internal rectal prolapse. Minimally invasive approaches are preferred as these make it easier to dissect between the vagina and the rectum in a narrow space. Also, it has been reported that minimally invasive approaches reduce the risk of complications and the length of hospital stay [27, 28]. There appears to be difference in the recurrence rates between laparoscopic and robotic rectopexy [26]. In another study, comparing open posterior rectopexy to laparoscopic rectopexy in 65 patients, the authors concluded that there was no significant difference between the outcomes achieved from the two procedures [29]. Recurrence rates vary from 0% to 33% in the reported literature [24]. The outcome of suture vs. mesh rectopexy [30] has also been reported to be similar. However, it must be remembered that these observations are based on small numbers and the quality of evidence to reach conclusions is poor.

In the Cochrane review of 15 randomized controlled trials consisting of 1007 patients [24], there was no difference in recurrence, complications, and quality of life between patients undergoing abdominal procedures and perineal surgery for rectal prolapse. Nearly a third of patients experience complications in the form of constipation, incontinence, and reduced rectal compliance following both procedures.
